# Epidemiological trend of suicide in north of Iran from 2011 to 2018 with a focus on joinpoint regression 

**DOI:** 10.22088/cjim.14.2.170

**Published:** 2023

**Authors:** Sharareh Zabihi Afroozi, Soraya Khafri, Ali Asghar Manouchehri, Mahasti Emami, Hajar Alizadeh, Sussan Moudi, Sara Khaleghi

**Affiliations:** 1 Department of Nursing, Babol University of Medical Sciences, Iran; 2 Social Determinants of Health Research center, Health Research Institute, Babol University of Medical Science, Babol, Iran; 3 Department of Internal Medicine, School of Medicine, Babol University of Medical Sciences, Iran; 4 School of Health, Iran University of Medical Sciences, Iran; 5 Department of Nursing, Babol University of Medical Sciences, Iran; 6 Student Research Committee, Babol University of Medical Science, Babol, Iran

**Keywords:** Epidemiology, Trend, Suicide, Joinpoint Regression, Iran

## Abstract

**Background:**

Deaths due to potential suicide are preventable and this phenomenon is costly for the health care system and contradicts the values and cultural standards of an Islamic country.

**Methods:**

This study is a retrospective study. The research population includes all cases of suicide during the years 2011-2018 who were referred to the emergency department of Babol hospitals. The results were analyzed using SPSS v.23 and Joinpoint Trend Analysis software 4.9.0.0 to identify significant changes in the temporal trends of the outbreak.

**Results:**

The highest percentage of suicides occurred in summer (27.8%), on Saturdays (13%) and at night (53%). A total of 1.9% of the cases were commit suicides (leading to death). The highest frequency of suicide was shown in 1397 (21.2%) and the lowest in 1392 (5.1%) and was more common in women (68.2% vs. 31.8%). Suicide-related deaths was higher in the second four years (63.5%), but suicide rate was significantly higher in the first four years (2011-2014) and the mortality rate due to suicide was higher in men than women.

**Conclusion:**

Suicide attempts were higher in women than men but the death rate was higher in men which means that men attempt suicide more seriously than women. The model also predicted that suicide rates would rise in the coming years. For this reason, this important issue, along with a detailed analysis of the roots of suicidal ideation and preventive measures, should be considered by health officials and social institutions.

Suicide is a non-accidental death that is mainly committed by the person him/herself with the aim of conscious or semi-conscious self-destruction. This act is not inadvertent and meaningless, but can be associated with unmet needs and despair (1). The conscious and purposeful act of suicide is often carried out by self-centered, anxious, aggressive, and incapable-of-socializing individuals with the goal of self-destruction, which is called "commit suicide"; however, if it is an act of suicide which does not lead to death, it is called "suicide attempt" or "unsuccessful suicide", which is usually used for those who plan their suicide in such a way that someone can prevent it, though in some cases no one can save them (2). According to the World Health Organization, by 2020, approximately 1.53 million people will die from suicide, and the rate of suicide in the world will increase by 10-20 times, with an average of one death per 20 seconds and one suicide attempt per 1-2 seconds (1). Suicide is the thirteenth cause of death in the world and the third cause of death in the age group of 15-34 years (3). 

Studies show that suicide rates vary from country to country, with the highest rates in the Scandinavian countries, Germany, Eastern Europe, Australia and Japan (25 per 100,000 people per year) and the lowest in Spain, Italy, Ireland, the Netherlands and Egypt (with 10 per 100,000 people per year). Suicide rate is low in most Muslim countries, so that in Kuwait the suicide rate is 0.1 per 100,000 people and in Pakistan less than 5 per 100,000 people. However, the suicide rate in Iran has increased in recent years and has reached 9.4 per 100,000 people (4), so that suicide and attempted suicide, especially among adolescents and young people in most parts of the country such as Kerman, Tabriz, Qazvin, Karaj, Shiraz, Kordkuy, Hamedan, Gilan, Masjed Soleyman, Dezful, Ahvaz, Eslamabad-e-Gharb and Mazandaran has increased (2).

According to suicide studies, there are many factors involved in suicide attempts. In this regard, the World Health Organization refers to psychological factors, mental and neurological (5). Age, gender, and marital status are other important social causes of suicide. Research has shown that the highest number of suicide attempts was in the age group of 15- 25 years (3). Due to the fact that according to official statistics, suicide statistics are increasing in our country, so this study aims to determine the incidence and predict the future incidence of suicide in patients referred to the emergency department of Babol hospitals. By providing this process to health officials and community social institutions, to enable them to prevent suicide. This statistical model identifies significant changes in a pattern over a period of time by considering the trend between joinpoint. One of the advantages of this method is the possibility of identifying the number and location of changes in the trend and estimating the average weekly percentage changes (AAPC) for each defined period joinpoints.

## Methods

This study was a retrospective study. The research population included all cases of suicide during the years 2011-2018, who were $\mathrm{corrected id}\times {\left(\frac{\mathrm{AAPCi}}{100}+1\right)}^{n}$ referred to the emergency department Babol hospitals a city in Mazandaran province. 

After going through the administrative process and obtaining a license from Babol University of Medical Sciences and making the necessary coordination with the officials of Babol hospitals, the available files were reviewed at the time of performing the project. This article is a research project approved by Babol University of Medical Sciences and Health Services in 2019 with the code IR.MUBABOL.REC.1399.353.

 Necessary information were extracted from the information registration forms available in the emergency part of the hospital and patients' medical records. The data collection tool, was a checklist had 14 questions, including: age, gender, level of education, marital status, place of residence (city/village), history of mental illness, history of physical illness, history of suicide attempt, history of suicide attempt in the family, method of suicide, motive for suicide, source of the report, result of the suicide attempt, and the season of committing suicide.

Finally, the results were statistically analyzed using SPSS software v. 23. First, the frequency of variables was calculated, then Chi-square and Fisher tests were used to investigate the relationship between these variables and the successful suicide variable. The multiple logistic regression model with Backward Stepwise (WALD) method was used to complete suicide rate. The factors and variables affecting commit suicide were entered to the model were; Sex, Age, Married status, Education, Place of residence, Season, Family history. Significance level was considered P<0.05. Then Joinpoint 4.7 software was used to identify change points (attachment points) and determine the trend between joinpoints and predict future suicide rates. The equation joinpoint regression model equation is defined as follows:

E[y |x] = β_0_ +β_1_ x +γ_1_ (x_i_ –τ_1_ )^+^+…+γ_n_ (x_i_ –τ_n_ )^+^+ ɛ_i_^k ^

As can be seen:

$\left\{\begin{array}{c} (x-\tau )+=(x-\tau )\mathrm{if}(x-\tau )>0\\ 0=\mathrm{otherwise} \end{array}\right.$

Where β_0_, β_1_, γ_1_,…. , γ_n_ are the regression coefficients and also τ_1_ <... <τ_k_ are the points of connection or the same points of refraction that k = 1, 2,… n and n <N, which are the *k*th of the unknown connection point. Also, the values of annual change percentage (APC) and average annual change percentage (AAPC) were used to compare the trend of decreasing or increasing the incidence and forecast in the future, the equation of which is to predict the future trend as follows. 

Update ID and AAPC is the average percentage of annual changes, the amount of which is obtained from the software update trend and n years of forecasting, which using this formula, the trend of future suicide will be predicted.

## Results

**Figure 1:**
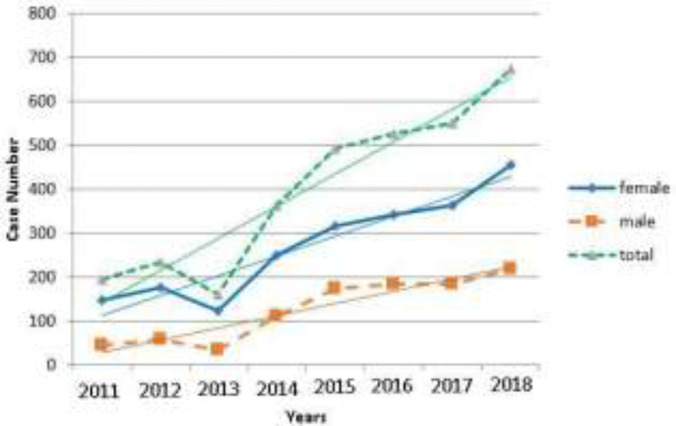
Suicide rate (by gender) in Babol city

**Figure 2:**
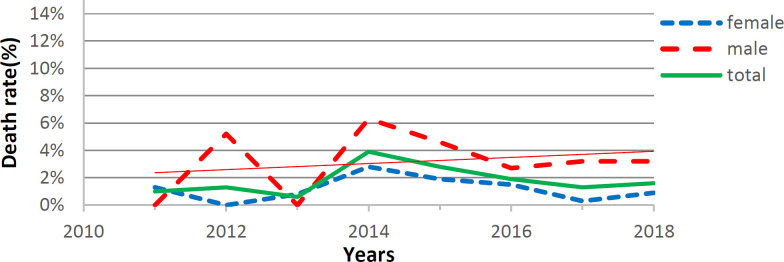
**S** **uccessful suicide rate (by gender) in Babol city**

The results of this study confirmed that a total of 3196 suicides were referred to the emergency department of Babol hospitals during the years 91-90. In (Figure 1) the highest frequency of suicide was shown in 1397 (21.2%) and the lowest in 1392 (5.1%). In this figure, the rate of people who attempted suicide based on gender were reported in women (68.2%) and men (31.8) that suicide was more common in women. In (Figure 2) when suicide-related deaths were examined, Suicide rate was higher in the second four years (63.5%), but suicide rate was significantly higher in the first four years (2011-2014). In this figure, it was observed that the mortality rate due to suicide is higher in men than women.

Also in this study, 1.9% of suicides (leading to death) were successful. The results of Table 1 show that the frequency of suicide attempts was higher in women (68.2%) but the rate of commit suicides in men was significantly higher (3.5%) (P<0.001). The age group under 40 years was the most common age (95.4%) and the age group over 68 years had the lowest (0.9%) cases of suicide attempt. The results of Fisher's exact test indicate a significant difference in cases of suicide attempts in different age groups (P<0.001) (Table 1). The mean of suicide age was 27.20±11.07 years. However, the rate of commit suicide was high (8.5%) in people over 68 years.

Table 1 indicates that the frequency of suicide attempts was high (66.4%) in urban areas, and the percentage of commit suicides in rural areas was significantly high (2.7%) (P=0.027). Also, the frequency of suicide attempts was high (37.5%) in people with high school education, and the rate of commit suicide was high in illiterate people (8.3%) (P<0.001). Suicide attempt was significantly higher in married people than single people (P<0.001). The results of the study indicate that a higher percentage (93.1%) of the individuals used the drug overdose method, but the percentage of commit suicide in other methods such as hanging, firearms, self-harm with a knife, etc. was higher, which indicated that there was a almost statistically significant relationship between suicide method and suicide outcome (P=0.059). Suicide attempt was significantly higher in married people than single people (P=0.035) (Table2). 

**Figure 3:**
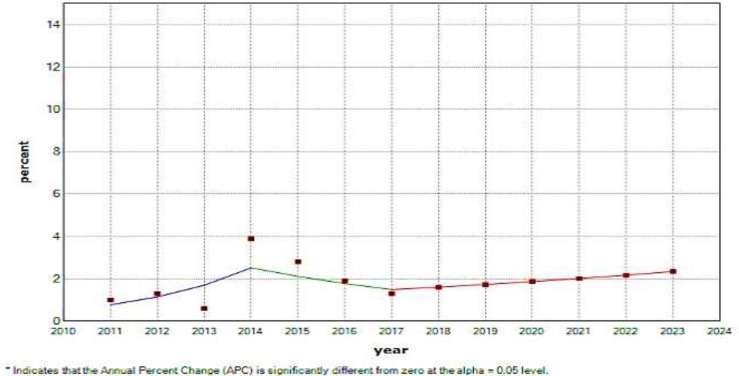
Trend of changes in the annual incidence of suicide in Iran during the years 2011-2018 and alsothe forecast during the years 2018-2023. In these models, the (APC) Annual percentage change in the incidence rate for positivecases indicates an increase in incidence cases. And in negative cases, we see a decrease in incidence and you can see it in figure 3

Figure (3) shows the data fit of the annual suicide incidence rate with two change points that only two break points were significant. annual Percentage changes (APC) Suicide incidence rate for the first plot in the year (2011-2014) equal to 3.48% with a confidence interval (2.109, 5.2) and for the second plot in the year (2014-2017) equal to -16 % With -confidence interval (-57.8, 66.9) and for forecasting in the third part in the year (2017-2023) is equal to 7.84% with confidence interval (-4 , 21.1) and also the average Annual percentage changes AAPC was calculated at 9.7%. In this model, it can be said that AAPC uses a single number to describe the average of APCs over a period of time. Estimated occurrence in this period is calculated on average with this ascending or descending slope. APC and AAPC can also be used as appropriate metrics to explain and summarize the process. Here AAPC = 9.7 reports that this year we are witnessing an increasing trend and every year compared to the previous year we will have an average increase of 9.7% in the city of Babel and in the future in constant conditions if no intervention with the same The trend is not done, we will see an average increase of 9.7% every year. 

The incidence of suicide for the second plot in the year (2014-2017) had the highest decrease of -16% and for the first plot in the year (2011-2014) the highest increase was equal to 3.48%. Use of segmented regression analysis enabled us to identify points in time when the trends changed significantly, generating quantitative hypotheses about changes in the behavior of the suicide among Babol city. The study of suicide motives revealed that the accelerating factor of 5% of suicides was family issues (dispute between husband and wife, and dispute between parents and children), 4.7% for unknown reasons, 1.4% due to quarrels, 0.7% due to mental illness, 0.4% due to love failure, and 0.3% due to other factors.

Regarding the percentage and frequency of history and status of physical and mental illness, it was found that most of the individuals did not have physical (P=0.395) and mental (P=0.356) illnesses, which was not statistically significant. The results of the study indicated that 72.8% of the individuals had no history of suicide attempt. Only 7.4% of the individuals reported a history of suicide among their family members, which is not statistically significant (P=0.607) (Table 2). According to Table 2, a higher percentage of suicides occurred in summer (28.2%), while in winter the rate of successful suicide attempts was higher (3.3%). Results show that 13% of the suicides occurred on Saturdays and 84.4% on non-holidays. Suicide attempts were higher at night (with a frequency of 53%) than at other times of the day. The results of logistic regression model in table3 are presented as adjusted and unadjusted for calculate the odds ratio (OR) and 95% confidence interval for commit suicide.

**Table 1.** Frequency distribution of demographic variables under study and their relationship with suicide outcome

**Variable**	**Suicide cases**	**P-value ** ^a^	**Successful outcome (death)**	**P-value ** ^b^
**Gender**				
Female	2180 (68.2)	P<0.001	26 (1.2)	P<0.001
Male	1016 (31.8)		36 (3.5)	
**Age category**				
Under 40 years	3048 (95.4)		50 (1.6)	
40-68 years	118 (3.7)	P<0.001	10 (8.5)	P<0.001
Over 68 years	30 (0.9)		2 (6.7)	
**Place of residence**				
City	2119 (66.4)	P<0.001	33 (1.6)	P=0.027
Village	1073 (33.6)		29 (2.7)	
**Education**				
Illiterate	48 (2.5)		4 (8.3)	
High school	951 (49.4)	P<0.001	6 (0.6)	P<0.001
Diploma	647 (33.6)		4 (0.6)	
University	278 (14.4)		2 (0.7)	
**Marital status**				
Single	1280 (41.1)		15 (1.2)	
Married	1864 (58.4)	P<0.001	46 (2.5)	P=0.035
Divorced - Widow	49 (1.5)		1 (2)	

The values in table are Number (percent).

a: the result of the nonparametric chi-square test; and b: the result of the chi-square test.

**Table 2:** Frequency distribution of other variables and their relationship with suicide outcome

**Variable**	**Suicide cases Number (percent)**	**P-value**	**Successful outcome (death) Number (percent)**	**P-value**
**Suicide method**				
medicine	2976 (93.1)	P<0.001	54 (1.8)	P=0.059
Other	220 (6.9)		8 (3.6)	
**Physical illness**				
Yes	243 (12.9)	P<0.001	4 (1.6)	P=0.395
No	1645 (87.1)		17 (1)	
**Mental illness**				
Yes	526 (25.6)	P<0.001	9 (1.7)	P=0.356
No	1526 (74.4)		18 (1.2)	
**History of suicide attempt**				
Yes	541 (27.2)	P<0.001	12 (2.2)	P=0.029
No	1448 (72.8)		14 (1)	
**Family history of suicide attempt**				
Yes	136 (7,4)	P<0.001	2 (1.5)	P=0.607
No	1690 (92.6)		17 (1)	
**Season**				
Spring	667 (20.9)	P<0.001	12 (1.8)	P=0.013
Summer	899 (28.2)		11 (1.2)	
Fall	838 (26.3)		13 (1.6)	
winter	788 (24,7)		26 (3.3)	

The values in table are Number (percent).

a: The result of the nonparametric chi-square test; and b: The result of the chi-square test

**Table 3:** Factors affecting successful suicide based on logistic regression model

**Variable**	**Crude OR (CI95%)**	**P-value**	**Adjusted OR (CI95%)**	**P-value**
**Gender**				
Female	Reference		Reference	
Male	4.37 (1.46 – 3.2)	0.008	4.2 (1.5 – 11.83)	0.007
**Education**				
Illiterate	Reference		Reference	
High school	0.117 (0.021 – 0.642)	0.013	0.083 (0.022 – 0.317)	0.000
Diploma	0.115 (0.019 – 0.698)	0.019	0.083 (0.019 – 0.352)	0.001
University	0.144 (0.018 – 1.12)	0.065	0.101 (0.017 – 0.583)	0.01
**Place of residence**				
City	Reference		Reference	
Village	3.64 (1.25 – 10.6)	0.018	3.1 (1.40 – 11.01)	0.000

## Discussion

The results of the present study regarding the epidemiological study of suicide in patients referred to the emergency department Babol hospitals confirmed that 3196 cases of suicide and attempted suicide were observed during 2011-2018, so that the suicide rate was higher in women than men, but the mortality rate in men was significantly higher. According to the results of the percentage of annual changes (APC) and the average percentage of annual changes (AAPC), it can be said that the trend of suicide has an upward trend until 2014 and a downward trend from 2014 to 2017, and in the forecast years with a upward trend have seen. In the observed trend, there has been a sharp decrease and increase i the incidence of suicide for 2013 and 2014, which has led to the observation of failure points. This sharp decrease and increase is somewhat unexpected, which can be due to a defect in data recording or inaccurate data collection methods. Therefore, focusing on the data obtained from the first and second parts can be more useful. The APC number for the forecast years was 7.84%. This number indicates that in the forecast years with a slope of 7.84% we will see an increase in suicide rates. This amount can be worrying and it is necessary to take preventive measures in this regard.

This result is consistent with the results of the studies by Mobasheri et al., Moradi et al., Jabbari Fard et al., Rafiei et al., Gorgi et al., Dadpour et al. in Mashhad city (2, 3, 6, 7, 8). The high rate of suicide attempts among women seems to be due to their economic dependence, lack of social support systems for women, emotional and relational problems, lack of self-confidence, and the dominance of patriarchy over family relationships. The high percentage of successful suicides in men is because of the methods they use, which are more violent than women’s methods. On the other hand, male personality is such that they consider their job, social relations and identity separately (6, 9). However, the results of the study conducted by Ansari et al. (2010) Ghalehiha et al. (2006), Moravveji et al. (2011), and Shao et al. (2013) are not consistent with the results of our study. This inconsistency may be due to differences in cultural and economic conditions of different regions of the world (10, 11, 12, 13).

The age range of suicide attempt in this study was 11-88 years, the highest rate of which was reported at the age of less than 40 years. In this regard, the results of Jabbari Fard et al.'s research showed that the highest suicide rate was in the age group of 15-34 years (6). In addition, the results of the study by Mobasheri et al. showed that the average age of suicide attempt was about 25 years (2), which indicates a higher suicide rate in the youth age group. The researchers declared that the reasons for the high prevalence of suicide in young people could be a disorder in their social relationships (mostly with their peers, spouse, parents), tendency to use drugs, medications, educational problems, impaired self-esteem, frustration and unemployment (3, 12). Although in our study suicide was more common in young people, the rate of commit suicide was higher in older people, which is consistent with the Shao’s study in Jiading and Shanghai (13).

 Regarding the manner of suicide attempt in the present study, 93.1% had used the drug overdose method. The results of the present study were consistent with most studies in the country and abroad (1, 2, 6, 7, 12, 14,15,16). In their study, Moravveji et al. stated that the prevalence of drug overdose method in Iran can be due to the availability of drugs, familiarity with various drugs, and the painlessness of this method. Drug overdose by those whose primary purpose in attempting suicide is to find a way to solve their problems (rather than death) can be another cause of the high prevalence of this method (12). These people may somehow want to draw the attention of others to their problem.

According to the results of this study, suicide attempts were significantly higher in married people than single people. In similar studies to our results (4, 7, 10, 14, 15) and vice versa, there was a high incidence of suicide in singles (1, 2, 6, 7, 10, 14). The reason for the higher suicide rate among married people in this city could be due to economic, cultural, and social problems, as well as family disputes. Regarding the accelerating factor of suicide attempts in this study, the highest motivation was family problems (dispute between husband and wife, and dispute between parents and children). The results of Jabbari Fard et al.'s study, too, confirmed that 62.2% of the reasons for suicide attempts were family disputes (6). In their study in Larestan and Gerash cities, Gorgi et al. stated that the most common reason for suicide attempt was family disputes (disputes and conflicts between adolescents and young people with their parents) (4). Also, Saberi Zafarghandi et al. stated that 48.2% of women and 19.8% of men expressed marital conflicts as the motive for suicide (15). Families are important socio-cultural centers and by enhancing the relationship and cohesion in the families, we can help reduce the suicide rate.

Based on the results of our study, most of the individuals did not have physical and mental illness, which was not statistically significant. The results of the study also indicated that 27.2% of the individuals had a history of suicide attempt. The results of other studies indicated that a small percentage of people had a history of mental illness (6, 7). In addition, in the study of Mobasheri et al. about 6%, in the study of Gorgi et al. (in the cities of Larestan and Gerash) 15.9%, and in the study of the Moravveji et al. 4% of the individuals had a history of attempted suicide (2, 4, 12). This is while in the statistics provided by the World Health Organization, this rate is stated as 20-30%. The researchers concluded that the lower frequency of previous suicide attempts in Iran may be due to cultural differences, more support and attention of the patient's family and relatives in order to solve problems, and secrecy of the individuals and their relatives. Considering that the act of suicide attempt is an alarm for fatal suicide in the future, it is necessary in the face of these patients not only to think about saving them, but also to provide psychiatric counseling to all patients (2). In this study, only 7.4% of suicide attempts were reported among family members, which is not statistically significant. In Jabbari Fard et al.’s study, 9%, and in the study of Ruengorn et al. 37.9% of cases of suicide history were reported in relatives (6, 16).

The study of the seasonal pattern of suicide during these eight years showed an increase in suicides in summer (28.2%) followed by autumn (26.3%). This result was consistent with the results of Khorshidi's study in Ilam city. According to this study, it can be acknowledged that special social conditions of the summer (such as failure to be accepted in universities, inability of families to provide education and welfare of their children, lack of effective programs to spend leisure time, unemployment and inadequate employment) can lead to despair and aggravate suicidal thoughts and behaviors (9). However, the results of the studies of Jabbari Fard, Ghalehiha, and Gorgi in Shiraz city showed an increase in suicides in the spring (4, 6, 7, 11), which may be due to differences in weather conditions and jobs in different seasons of year, as well as the sample number. Meanwhile, the results of the present study revealed that the rate of commit suicide attempt is higher in winter (3.3%) and then in spring (1.8%), which is somewhat consistent with the results of Jabbari Fard et al. In their study, they stated that the highest rate of successful suicide is in autumn and then in spring (6). In addition, the results of Khorshidi’s study indicated an increased risk of suicide leading to death in winter (9). The lack of uniform distribution of suicide in different seasons as shown in this study and other studies may indicate the influence of season-related factors on the incidence of suicide.

Regarding the relationship between suicide and days of the week, the research results indicated that 13% of the suicides occurred on Saturdays (the first working day of the week in Iran). Also, the results of the study of Ghalehiha et al. showed that on Thursdays and Saturdays, the highest suicide rates were 15.8% and 15.5%, respectively (11). Also, the results of some studies confirm that in developed western countries, the highest number of suicides occur on Monday (the first working day of the week) and the lowest number of suicides occur on Saturday (the first day of weekly holiday) (19), which is consistent with the present study. Regarding the time of suicide, the highest suicide rate occurred at night with a frequency of 53%, which was consistent with the study of Ghalehiha (11). Perhaps the reason for this is that people have free time at night and they focus more on their daily problems. According to studies, these results appear to be important in preventing suicide and raising awareness among families. One of the strengths of the present study is examining suicide trend during 2011–2018. One of the limitations of the study was the incompleteness of some information in the files, such as not registering the season of committing suicide in a number of cases. 

The overall results of this study showed that the highest rate of suicide occurred in married women, people under 40 years old, people with high school education, urban dwellers and in summer. According to the results obtained from the scattered regression model, it can be said that the suicide pattern has been declining since 2014, but for the predicted years with a slight slope, we see an upward trend that requires further investigation and preventive measures. Results of this study it showed that women commit suicide more than men and men have a higher mortality rate, which means that men commit suicide more seriously than women. Therefore, paying attention to these factors can play an effective role in reducing this phenomenon in society. In addition, it is necessary to plan and provide solutions such as the development of counseling, educational, welfare and social services by health officials and social institutions. However, on average, the data related to the various variables studied in this study had missing data. Therefore, it is recommended to create a systematic program to record all the characteristics of people who attempt suicide and factors related to suicide.
